# Development and validation of the RACER (Readiness for Adult Care in Rheumatology) transition instrument in youth with juvenile idiopathic arthritis

**DOI:** 10.1186/s12969-021-00579-1

**Published:** 2021-06-05

**Authors:** Lynn Spiegel, Lori Tucker, Karen Watanabe Duffy, Chitra Lalloo, Amos Hundert, Josiane Bourre-Tessier, Elizabeth Hazel, Nadia Luca, Dianne Mosher, Cynthia Nguyen, Elizabeth Stringer, Charles Victor, Jennifer Stinson

**Affiliations:** 1 Division of Rheumatology, Department of Pediatrics, Hospital for Sick Children, University of Toronto, Toronto, Canada; 2 Division of Rheumatology, BC Children’s Hospital, University of British Columbia, Vancouver, Canada; 3 Division of Rheumatology, Children’s Hospital of Eastern Ontario (CHEO), University of Ottawa, Ottawa, Canada; 4 Child Health Evaluative Sciences, The Hospital for Sick Children, Toronto, Canada; 5 Institute of Health Policy, Management & Evaluation, University of Toronto, Toronto, Canada; 6 Division of Rheumatology, Centre hospitalier de l’Université de Montréal, Montreal, Quebec Canada; 7 McGill University Health Centre, Montréal, Quebec Canada; 8 Section of Pediatric Rheumatology, Department of Pediatrics, Alberta Children’s Hospital, University of Calgary, Calgary, Canada; 9 University of Calgary and Cumming School of Medicine, Calgary, Canada; 10 Department of Pediatrics, IWK Health, Dalhousie University, Halifax, Canada; 11 Institute for Clinical Evaluative Sciences, Toronto, Canada; 12 Lawrence S. Bloomberg Faculty of Nursing, University of Toronto, Toronto, Canada; 13 The Hospital for Sick Children Peter Gilgan Centre for Research and Learning, 686 Bay Street, Room 069715, ON M5G 0A4 Toronto, Canada

## Abstract

**Background:**

Current evidence suggests that many adolescents with juvenile idiopathic arthritis (JIA) do not successfully transfer to adult care, which can result in adverse health outcomes. Although a growing number of clinical programs have been designed to support healthcare transition, there is a lack of psychometrically sound instruments to evaluate their impact on development of transition-related knowledge and skills in youth with JIA. The purpose of this study was to develop and validate RACER (Readiness for Adult Care in Rheumatology), a self-administered instrument designed to measure stages of readiness for key transition-related skills in adolescents with JIA.

**Methods:**

A phased approach was used to develop and evaluate the validity and reliability of RACER. Phase 1 A was a consensus conference with 19 key stakeholders to inform instrument domains and items. Phase 1B determined initial content validity using a sample of 30 adolescents with JIA and 15 clinical and research experts. Finally, Phase 2 was a prospective cohort study with repeated measures to evaluate the internal consistency, test-retest reliability, construct validity and responsiveness of the instrument within a sample of adolescents with JIA.

**Results:**

In Phase 1 A, initial item generation yielded a total of 242 items across six domains from the consensus conference, which was subsequently reduced to a 32-item instrument. Phase 1B established the content validity of the instrument in adolescents with JIA. In the Phase 2 study, with a sample of 96 adolescents, the RACER instrument exhibited good internal consistency in five of its six subscales (Cronbach’s α > 0.7), and strong test-retest reliability between the first two administrations (ICC = 0.83). It also showed robust convergent validity by highly correlating with measures of self-management (SMSAG, rho = 0.73) and transition (TRANSITION-Q, rho = 0.76). The RACER was not correlated with unrelated measures (discriminant validity; PedsQL, rho = 0.14). The RACER scores increased significantly over time as expected, supporting measure responsiveness.

**Conclusions:**

The RACER is a reliable and valid instrument which is sensitive to change for assessing transition readiness in adolescents with JIA.

## Background

Juvenile idiopathic arthritis (JIA) is a common chronic childhood illness that can negatively impact quality of life [1, 2]. Disease management is complex and often requires multiple therapies over long periods of time [3]. As patients move into adult-oriented healthcare systems, adolescents are expected to take greater responsibility for disease management as they assume increasing independence and autonomy [4]. Successful health care transition requires that adolescents acquire skills in self-care, healthcare decision-making, and self-advocacy that will prepare them to take more responsibility for their health and healthcare needs [5–7]. A study done by Hazel et al. showed that many adolescents with JIA (up to 52 %) did not successfully transfer to adult care. An unsuccessful transition was defined as having not made initial contact with the designated adult rheumatologist, ongoing follow up in the first 2 years after transfer, and/or not demonstrating the self-management skills that are necessary to be successful in the adult health care setting [8–10]. Failure to transition to ongoing care of an adult rheumatologist can result in significant future health consequences such as joint damage, loss of function, and increased hospitalizations [4, 11, 12]. As well, poorly planned transition is associated with an increased risk of treatment non-adherence, and with subsequent negative consequences in terms of social, educational, and vocational outcomes [3, 13, 14].

To address the challenges of transition, many pediatric rheumatology centers have developed specialized programs that help adolescents develop transition-related skills [15, 16]. National and international position statements and policy documents outline the strong need to evaluate the impact of transition programs [17, 18]. Despite the increasing number of transition programs, there is a lack of evidence of their effectiveness [19, 20]. The lack of a valid and patient-centered instrument to assess transition-related knowledge and skills in adolescents with JIA is one factor in the difficulty in assessing transition program efficacy.

Our research group published a systematic review and critical appraisal of existing transition readiness and transfer satisfaction measures [19]. We identified seven readiness and seven satisfaction measures for evaluating rheumatology transition programs, none of which had well established reliability and validity [19]. Most measures were developed ad-hoc by an investigator group with minimal to no evidence of reliability and/or validity based on Cohen’s criteria and the COnsensus-based Standards for the selection of health status Measurement INstruments (COSMIN) checklist [21, 22]. Furthermore, most studies did not outline whether adolescents were involved in the development and testing of these patient-reported outcome measures.

 For transition to adult-based care to be successful, it is critical that adolescents are prepared to assume the increasing responsibility of their health care. A valid and reliable transition readiness measure provides the ability for clinicians and researchers to: (1) track the progress of adolescents throughout the transition process, (2) pinpoint the relative stage of ‘transition readiness’ for individual patients in order to identify adolescents at-risk for poor transition and tailor interventions accordingly, (3) educate and inform pediatric and adult healthcare providers about the need for personalized intervention related to education and self-management skills training, and (4) objectively compare the effectiveness of different transition programs across populations.

To address this need, our research goals were to develop and evaluate the RACER (Readiness for Adult Care in Rheumatology), a self-administered instrument designed to measure stages of readiness for key transition-related skills. This research project was completed in several phases: In Phase 1 A, we aimed to achieve consensus among experts in transition care and rheumatology on key domains and items for a transition readiness measure in adolescents with JIA. In phase 1B, we aimed to determine initial content validity of the instrument in a sample of adolescents with JIA, clinicians, and researchers. Finally, in Phase 2, we aimed to prospectively evaluate the reliability and validity of the RACER as well as its ease of use in a cohort of adolescents with JIA aged 16 to 20 years followed for a 28 month period. Specifically, we evaluated the RACER for internal consistency, test-retest reliability, construct validity and responsiveness.

## Methods

### Phase 1 A: Consensus conference

In the fall of 2010, we conducted a two-day conference to develop consensus among key stakeholders from across North America regarding the domains and items that should be included in the RACER. Clinical and research experts in the fields of adolescent medicine, pediatric and adult rheumatology, psychology, and health measurement, as well as young adults with JIA and parents of children with JIA participated.

A voting process using the nominal group technique, a structured meeting with round robin voting, was used to facilitate consensus among experts [23]. Discussions were used to resolve disagreements concerning definition and conceptualization of transition readiness, as well as the domains, items, scaling, and wording of the instrument. Domains and items that achieved consensus (agreed to by ≥ 75 % of participants) were included in the initial RACER draft. The resulting instrument was originally developed in English and then subsequently translated into French. All subsequent phases of the study were conducted concurrently with the English and French versions.

### Phase 1B: Initial content validation

A descriptive study design was used to determine comprehensiveness, relevance, and understanding of the RACER. Clinicians and researchers with experience in adolescent rheumatic medicine who were not part of the consensus conference were invited to test the content validity of the instrument. A purposive sample of Adolescents (aged 12–18 years) and one of their parents were recruited from Pediatric rheumatology clinics at two major Canadian pediatric rheumatology centres.

Participants were asked to rate the relative importance of each domain and item. They were also asked about the clarity of the content, meaning, wording, and intelligibility of items and whether they felt that there were any missing domains, and / or items. The importance of each domain and item to the process of health care transition was rated on a 5-point Likert scale ranging from 1 (‘not important’) to 5 (‘highly important’), and the content validity ratio (CVR) was computed [24]. The content validity ratio varies between one and negative one, with a higher score indicating greater agreement among the respondents on the necessity of an item. If more than half of the respondents rated an item as being important, (average score of 4 or above), the content validity ratio was positive, the item was considered to be relevant, and was included [24].

### Phase 2: Validation

#### Participants

A prospective cohort study with repeated measures was completed over a 28-month period to evaluate internal consistency, test-retest reliability, construct validity and responsiveness. A sample of English and French speaking adolescents with JIA receiving standard care were recruited from the active treatment populations of six Canadian pediatric and adult tertiary care rheumatology centers. Adolescents were eligible for study inclusion if they were: aged 16–20 years, diagnosed with JIA and were under care by a pediatric rheumatologist, able to read and speak English or French, and willing to complete online self-report measures. Adolescents were not eligible if they had moderate to severe cognitive impairments or major psychiatric co-morbid illnesses that precluded them from completing the online self-report measures, as determined per their healthcare provider.

#### Sample size

Our recruitment goal was 84 English-speaking and 84 French-speaking adolescents. The calculation assumed 80 % power to detect differences of 0.2 between two ICC coefficients assuming type I error of 0.05 and moderate reliability to evaluate test-retest reliability. For construct validity testing, this sample size would achieve 80 % power to detect difference of 0.225 in Pearson’s correlation coefficient assuming type I error of 0.05 and null Pearson’s correlation coefficient of 0.50. Finally, to evaluate responsiveness this sample size would achieve 80 % power to detect small effect size of 0.33 or larger assuming type I error of 0.05 and modest with-in subject correlation of 0.5.

#### Procedure

The RACER questionnaire was completed at five time points over 18 months (baseline, 2 weeks, 6, 12, and 18 months post baseline visits, referred to as T1 through T5 respectively). Additional data collected at baseline included: patient demographics, disease characteristics, self-management skills, and health related quality of life. JIA diagnosis was obtained from the patient chart using the ILAR classification criteria [25]. A baseline global score of JIA disease severity was obtained from the physician, with scores ranging from 0 to 10 and higher scores indicating greater disease severity [26]. All measures were completed online by participants using Research Electronic Data Capture (REDCap). Participants were sent the electronic measures directly, with no clinician or family involvement in asking or answering the questions.

## Measures

### RACER

The 32-item RACER self-report instrument is designed to measure stages of readiness for key transition-related skills, and is organized into the following domains: General Knowledge (8 items), Knowledge About Medications (3 items), Planning For Adult Life (5 items), Managing Your Health Condition (6 items), Standing Up For Yourself (6-items), and Knowing How to Get Around the Healthcare System (4-items). The Knowledge domain responses were “yes”/”no”, with a “not applicable” option for Medication Knowledge questions. All other domain responses used a four-point Likert scale ranging from “I do not know how but I want to learn” to “I always do this when I need to”. A summative sub-score was calculated for each individual domain and then rescaled to range from 0 to 100. The equally weighted average across each domain was then calculated to create a total score (0-100). Respondents who indicated “not applicable” for the medication subscale, had their average score calculated out of 5 domains, whereas the scores for those respondents who were on medication were calculated out of 6 domains. Higher scores indicate greater transition readiness. The subsequent three measures were used to evaluate RACER construct validity.

### Self-Management Skills Assessment Guide (SMSAG)

The SMSAG is a 21-item validated questionnaire that assesses patient awareness of their health condition and ability to make decisions relevant to their healthcare needs [27]. All items are rated on a five-point Likert scale as follows: 1 = strongly disagree, 2 = disagree, 3 = neither disagree or agree, 4 = agree, 5 = strongly agree. Total score was calculated as a mean of all item responses, with a maximum value of 105.

### TRANSITION-Q

The TRANSITION-Q 14-item scale is a generic scale that measures self-management skills in health and healthcare in adolescents (aged 12–18 years) with chronic health conditions [28]. The total scale score was used, ranging from 0 (poor self-management) to 28 (excellent self-management).

### PedsQL 3.0 arthritis module

The PedsQL has 22 items and evaluates the severity of perceived problems with disease symptoms, daily activity limitations, treatments, worry/anxiety, and communication [29]. The total scale score was used for analyses, which ranges from 0 (poorest quality of life) to 100 (excellent quality of life).

### Data analysis

Data were analyzed using R version 3.4.2. Descriptive statistics were used to describe the cohort at baseline. Rates of accrual, dropout, compliance, and missing data were calculated. Demographic characteristics and baseline test scores were compared between participants that were lost to follow-up and those that completed the study.

The following psychometric properties of the RACER were assessed. Cronbach alpha was used to measure internal consistency for each domain. Alpha values of 0.70–0.95 were used as a cut off to indicate adequate internal consistency [30]. We then examined the relationship of each of the items with their respective domain score using a Pearson correlation, hypothesizing that each item would have a moderate to high correlation coefficient (rho ≥ 0.3). The Shrout and Fleiss ICC [31] and 95 % confidence intervals were used to measure test-retest reliability between the first two administrations of the RACER (Baseline and 2-weeks). We used a cut-off of 0.8 or greater to demonstrate strong test-retest reliability. We hypothesized a 95 % confidence interval width of 0.40 around ICCs in the range of 0.75. Pearson’s correlation coefficients (rho) and their 95 % confidence intervals were used to measure construct validity by comparing baseline RACER scores to SMSAG, TRANSITION-Q and PedsQL scores. It was hypothesized that the SMSAG and TRANSITION-Q would be highly correlated to the RACER scores (rho ≥ 0.75), indicating evidence of convergent validity. Furthermore, we expected to observe a low correlation (rho < 0.25) between the RACER and the PedsQL, indicating evidence of divergent validity. The responsiveness of the RACER to changes in transition readiness was evaluated using the standardized response means (SRMs): [SRM = absolute mean (test2 –test1)/SDdiff]. The SRM was hypothesized to be low (< 0.5) from T2-T4 and moderate (0.5–0.75) from T1-T5 [32]. Linear mixed models with participant as the random factor, and time as the fixed factor, were also used to evaluate the responsiveness of the RACER to change over time. Beta coefficients with standard error, 95 % confidence interval, and corresponding p-values were used to evaluate the change in subscale scores at the various study time points.

## Results

### Phase 1 A: Consensus conference

A total of 19 clinical and research experts, two young adults with JIA, and one parent of a child with JIA participated in the consensus conference. Six domains were generated: (1) General Knowledge; (2) Medication Knowledge; (3) Planning for Adult Life; (4) Managing your Health Condition; (5) Standing Up for Yourself; and (6) Knowing How to Get Around the Healthcare System. A total of 242 items were generated across domains and were subsequently reduced to 32 key items.

### Phase 1B: Content validation

A sample of 15 clinical and research experts participated in content validation along with 30 adolescents with JIA. Adolescents were 60 % English speaking (*n* = 18), 73 % female (*n* = 22) and had an average age of 14.5 (SD = 1.7) years. Based upon the content validity ratios and respondent feedback, minor changes were implemented (see Table 1). Results supported the comprehensiveness, relevance, and understanding of the RACER in adolescents with JIA. The revised RACER consisted of 32 self-report items, organized into the 6 domains noted above.

**Table 1:** Modifications to the RACER based on pilot testing

Reason	Tested RACER Item	Revised RACER Item
Redundancy with existing item	Do you talk about your health condition with your teachers and/or your boss?	Do you know how to deal with your emotions?
Improves understanding	Do you make plans for medical care when you travel?	Do you make the necessary plans to manage your medical condition when you leave home (i.e., for travel, school, or a job)?
Improves understanding	Do you recognize the signs that you are getting sick?	Do you know how to recognize the signs that your medical condition is getting worse?

### Phase 2: Validation

#### Sample

Ninety-six English-speaking and 26 French-speaking participants participated across the eight sites. Due to French-speaking sample enrollment below the a priori sample size (*N* = 84), the study was not adequately powered to examine the French RACER instrument. Demographic and disease characteristics for the English-speaking adolescent participants are summarized in Table 2 with scores on the RACER subscales at all time points presented in Table 3. At baseline English participants completed the RACER in 4.7 min on average (SD = 4.4).

**Table 2:** Demographic and Clinical Characteristics of the Study Participants

Characteristic	*N* = 96
Age in years, mean (range)	17.5 (15.7–20.6)
Recruitment centre	
Pediatric	82 (85 %)
Adult	14 (15 %)
Global disease severity (0–10), mean (range)	1.32 (0–8)
Sex, n (%)	
Male	31 (32)
Female	65 (68)
Diagnosis, n (%) Arthritis sub-type	
Enthesitis Related Arthritis	21 (22)
Oligo persistent	15 (16)
Oligo extended	8 (8)
Poly RF negative	15 (16)
Poly RF positive	4 (4)
Psoriatic	11 (11)
Systemic	11 (11)
Unclassified	3 (3)
Undifferentiated	2 (1)
Other	5 (5)
Unknown	1 (1)

**Table 3:** The RACER instrument scores at each study time point

Domain	Score, mean (SD)				
	T1 (baseline)	T2 (2 weeks)	T3 (6 months)	T4 (12 months)	T5 (18 months)
	*n* = 91	*n* = 93	*n* = 79	*n* = 77	*N* = 77
General Knowledge	81.8 (16.7)	81.2 (17.8)	87.2 (15.0)	91.0 (12.9)	90.1 (17.0)
Knowledge About Medications	69.5 (35.8)	68.5 (37.9)	69.2 (36.1)	74.5 (35.4)	75.3 (33.9)
Planning for Adult Life	70.2 (20.8)	69 0.8 (22.5)	74.1 (20.2)	80.7 (17.8)	83.4 (19.5)
Managing Your Health Condition	79.6 (17.2)	77.7 (20.7)	83.1 (16.2)	84.8 (17.0)	87.4 (17.5)
Standing Up for Yourself	73.5 (22.7)	74.4 (23.6)	77.6 (22.9)	82.1 (19.8)	84.1 (20.4)
Knowing How to Get Around the Healthcare System	61.3 (30.5)	63.8 (32.2)	66.5 (30.5)	75.6 (25.3)	80.3 (23.8)
Total	73.0 (16.9)	72.6 (18.9)	76.3 (17.1)	81.4 (14.6)	83.4 (14.7)

#### Missing data

Ninety-six (100 %) of the English-language participants completed their baseline and were enrolled in the study. Those missing 50 % or more items within at least one RACER domain were excluded from analysis of psychometric properties at that time point. Overall missing data were as follows: T1 (*n* = 5, 5 %) T2 (*n *= 3, 3 %), T3 (*n* = 17, 18 %), T4 (*n* = 19, 20 %), T5 (*n* = 19, 20 %).

### Psychometric properties

#### Internal consistency

All but one domain met the criteria for internal consistency (General Knowledge a = 0.63). Medication Knowledge (0.92), Planning for Adult Life (0.75), Managing Your Health Condition (0.76), Speaking Up for Yourself (0.85), Knowing How to Get Around the Healthcare System (0.86) all met the threshold of 0.70. This finding indicates that the items were correctly aligned within their respective domains. Similarly, the correlations of each of the items within each domain were moderately to strongly correlated to one another (r ≥ 0.3) **(**Table 4**)**.

**Table 4:** Internal Consistency measures (raw variables) of items and subscales of the RACER instrument measured at baseline

Item	R	Alpha (95 % CI)
**General Knowledge**		
K1	0.57	0.63 (0.55–0.65)
K2	0.3	
K3	0.42	
K4	0.5	
K5	0.51	
K6	0.33	
K7	0.71	
K8	0.67	
**Knowledge About Medications**		
K9	0.93	0.92 (0.91–0.93)
K10	0.92	
K11	0.94	
**Planning for Adult Life**		
P1	0.69	0.75 (0.71–0.78)
P2	0.72	
P3	0.73	
P4	0.68	
P5	0.68	
**Managing Your Health Condition**		
M1	0.72	0.76 (0.72–0.79)
M2	0.65	
M3	0.79	
M4	0.73	
M5	0.51	
M6	0.65	
**Standing Up for Yourself**		
S1	0.7	0.85 (0.83–0.87)
S2	0.69	
S3	0.78	
S4	0.83	
S5	0.82	
S6	0.76	
**Knowing How to Get Around the Healthcare System**		
HS1	0.79	0.86(0.84–0.88)
HS2	0.8	
HS3	0.86	
HS4	0.82	

#### Test retest reliability

The ICC for the RACER instrument compared at T1 and T2 was 0.83 (95 % CI 0.79–0.86), exceeding the defined cutoff of 0.80.

#### Construct validity

The RACER and the SMSAG were strongly correlated with each other (rho = 0.73). Similarly, the RACER and Transition-Q were positively correlated with one another (rho = 0.76). As hypothesized, the RACER and PEDSQL were weakly correlated (rho = 0.15) **(**Table 5**)**.

**Table 5:** Measurements of construct validity at baseline for the RACER instrument

Questionnaires	Test	Pearson correlation (range)	N
RACER VS SMSAG	Convergent Validity	0.73 (0.62–0.82)	85
RACER VS TQ	Convergent Validity	0.76 (0.65–0.83)	88
RACER VS PEDS-QL	Discriminant Validity	0.15 (-0.062–0.34)	90

#### Responsiveness

The SRMs for the RACER at 2 weeks post-baseline were trivial (0.048), small at 24 weeks (0.28) and moderate at 48 (0.62) and 72 (0.73) weeks post baseline, results which are in line with the hypotheses. As hypothesized, there was no significant increase in the RACER score at two weeks (T2) compared to baseline. The regression model demonstrated that the RACER score increased significantly from baseline compared to T3 through T5. **(**Table 6**)**.

**Table 6:** Linear mixed-model parameters for repeated measures of the RACER instrument (*N* = 96)

Time points	Beta coefficient	95 % Confidence interval	*P*-value
**T1 vs. T2**	-0.22	-2.53–2.08	0.850
**T1 vs. T3**	3.69	1.14–6.24	0.005
**T1 vs. T4**	9.11	6.37–11.85	< 0.001
**T1 vs. T5**	10.9	7.95–13.93	< 0.001

## Discussion

Through a phased approach we developed and validated the psychometric properties of a new tool for the assessment of transition readiness in adolescents with JIA. A consensus conference with key stakeholders to inform items and domains of the RACER, helped to ensure that RACER targets key aspects of transition readiness, and is relevant to patients, parents, and clinicians and researchers. Validation was conducted in a sample which included all JIA subtypes and was broadly reflective of the JIA population overall, with Oligoarthritis being the most common [33]. Compared to other related questionnaires, the RACER item generation and creation underwent a theoretically informed and rigorous development process. Overall, the RACER demonstrates strong internal consistency, construct validity, significant responsiveness, and satisfactory test-retest reliability.

The internal consistency for five out of the six RACER subscales met the 0.70 threshold, indicating that the items within these domains are highly correlated and measure the same construct. Specifically, the RACER exhibited similar or greater values of internal consistency compared to other validated questionnaires of transition readiness, for example, the TRAQ “Self-Management” subscale (α = 0.92) and “Self-Advocacy” subscale (α = 0.82) [34]. The domain “Knowledge” had an alpha slightly under the threshold (a = 0.63). This finding may be because knowledge related to one’s rheumatic condition are also associated with concepts in other domains. For example, the question “Do you know the differences between the child and adult health care systems?” may relate more to concepts in the Health Systems domain as compared to those in General Knowledge. In future studies, researchers should employ tests of structural validity to assess the underlying structure of each domain.

The RACER instrument exhibited robust convergent and discriminant validity. As expected, responses on the RACER instrument were highly correlated to measures of self-management (SMASAG) and transition (TRANSITION-Q). The RACER instrument was not related to constructs measuring quality of life (PedsQL). We also observed strong and significant responsiveness of the RACER instrument over the study period. These results are in line with previous measures of response in transition measures such as the TRANSITION-Q, I am ON TRAC, and TRAQ [28, 35, 36]. This finding is important with respect to facilitating transition from one clinic visit to another, and suggests the possible use of the RACER instrument as a pre- and post-test assessment for transition and self-management interventions in a developmentally appropriate fashion [37, 38].

Most common transitions questionnaires such as the Transition-Q and TRAQ are generally validated and designed for a broader population of pediatric health conditions. While this allows for comparison across other populations, the broader questionnaires do not evaluate transition concepts specific to JIA, limiting their clinical utility within this population. To our knowledge, the RACER is the only JIA focused instrument that demonstrates significant internal consistency and test-retest reliability, robust construct validity, and a high level of responsiveness specifically in a pediatric rheumatology setting.

## Limitations and next steps

The study was not powered to examine the validity of the French RACER instrument. Future research with a larger sample size will be required. The study evaluated self-report of the RACER instrument. Further research could examine the use of parent proxy completion for individuals with cognitive impairments. Additionally, there was no specific transition intervention delivered across the sites and no disease or health care utilization outcomes were captured. Further research in a prospective trial could assess if the RACER instrument can discriminate between participants undergoing a given transition readiness program compared to controls. Finally, although testing was done in the JIA population, we hope to generalize the use of this instrument to other rheumatic conditions, with future research evaluating RACER in other populations to assess the generalizability and validity of the instrument. As a next step, we will use the RACER annually in JIA clinics for youth aged 16–18 to assess transition readiness.

## Conclusions

The RACER measure has been validated as a clinical instrument to monitor transition readiness in youth diagnosed with JIA. We determined that the RACER is reliable, valid, and responsive to change in adolescents with JIA. This instrument will help to assess adolescents’ progress throughout transition and their readiness to move to adult care. Clinically, it may also be used to help identify adolescents who are at-risk for poor transition, and promote responsive modification of their care. The RACER is a valuable instrument that can be used in research and clinical care. It can also be used to evaluate the effectiveness of transition programs developed to help adolescents move successfully to adult health care. Ultimately, the information it provides should improve the health, and health-related outcomes in adolescents and adults with JIA.

## Data Availability

The full dataset is not publicly available due to participant privacy and confidentiality. Limited data may be made available upon request to the corresponding author.

## Abbreviations

**COSMIN**: COnsensus-based Standards for the selection of health status Measurement INstruments
**CVR**: Content validity ratio
**ICC**: Intra class correlation
**JIA**: Juvenile idiopathic arthritis
**PedsQL**: Pediatric Quality of Life Inventory
**RACER**: Readiness for Adult Care in Rheumatology
**REDCap**: Research Electronic Data Capture
**SMSAG**: Self-Management Skills Assessment Guide
**SRM**: Standardized response mean
